# *ZmMed31–ZmSIG2A* Coordinates ROS Homeostasis and LRR-RLK Signaling to Regulate Root Development

**DOI:** 10.3390/plants15071057

**Published:** 2026-03-30

**Authors:** Dan Jiang, Shengwei Guo, Xin Yuan, Sheng Zhang, Yuxin Zhang, Yuqi Ning, Fujian Qu, Qunkai Niu, Moju Cao

**Affiliations:** 1 State Key Laboratory of Crop Gene Exploration and Utilization in Southwest China, Maize Research Institute, Sichuan Agricultural University, Chengdu 611130, China; njsgskl@163.com (D.J.); guo8871@sina.com (S.G.); scauymsyx@163.com (X.Y.); 19862023505@163.com (S.Z.); 18637706863@163.com (Y.Z.); nyuqi_77@163.com (Y.N.); 13654658794@163.com (F.Q.); 2 Chengdu Agricultural College, Chengdu 611130, China

**Keywords:** maize (*Zea mays* L.), *ZmSIG2A*, root development, reactive oxygen species (ROS) homeostasis, antioxidant enzymes, LRR-RLK

## Abstract

*ZmSIG2A* is a nuclear-encoded plastid sigma factor 2A in maize (*Zea mays* L.) that is essential for plastid gene transcription and chloroplast biogenesis. As a key regulator of chloroplast development and function, *ZmSIG2A* may also contribute to the coordination of plant growth and environmental adaptation; however, its roles in root development and stress responses remain largely unclear. We compared two *ZmSIG2A* mutants, *eal1-1* (hypomorphic) and *ems110* (nonsense). *eal1-1* had increased root number and longer roots, while *ems110* had normal root number but shorter roots and failed to mature. The *zmsig2a^Val480del^* transcript was upregulated in *eal1-1*, and the root-promoting effect of *OsSIG2A* in rice suggests a conserved role in monocot root growth. DAP-seq indicated that *zmsig2a^Val480del^* targets are involved in metabolism, transport, signaling, and antioxidants, with Chr4 peak clustering near multiple LRR-RLKs, suggesting a *ZmSIG2A*–LRR-RLK module in root development and stress integration. Physiologically, *eal1-1* showed increased antioxidant enzyme activities and reduced MDA, indicating enhanced ROS scavenging, while *ems110* exhibited decreased enzyme activities and elevated MDA, indicating compromised ROS detoxification. Upstream, Y1H and dual-luciferase assays demonstrated that the Mediator subunit *ZmMed31* positively regulates transcription from the *ZmSIG2A* promoter. Given Mediator’s role in bridging transcription factors and the core transcriptional machinery, *ZmMed31* likely links hormone-responsive transcription factors to the *ZmSIG2A* regulatory network. Collectively, we propose a stress-responsive *ZmMed31*–*ZmSIG2A*–LRR-RLK module that underpins maize root development and drought adaptation, offering mechanistic insight and potential targets for stress-resilient breeding.

## 1. Introduction

Plant σ factors are highly homologous to cyanobacterial σ factors. The endosymbiotic theory proposes that plant σ factors originated from the endosymbiotic event between a cyanobacterial ancestor and the progenitor of plants, and the continuity between chloroplast transcriptional machinery and bacterial transcription provides further theoretical support for this hypothesis [1]. Functionally, chloroplast σ factors resemble their bacterial counterparts, acting as accessory factors of the plastid-encoded RNA polymerase (PEP) to confer promoter recognition and initiate transcription. From an evolutionary perspective, this reflects the bacterial legacy retained in the chloroplast transcription system and reinforces the endosymbiotic origin of plastids. During plant evolution, the σ-factor family underwent gene duplication and functional divergence, resulting in a pattern in which conservation and specialization coexist, enabling plastid gene transcription to be more finely tuned to developmental programs and environmental cues [2]. Beyond regulating plastid gene expression, σ factors are deeply involved in photosynthetic regulation and stress responses. In *Arabidopsis*, *AtSIG1*–*AtSIG6* display distinct promoter selectivities and therefore partition the regulation of different plastid gene modules [3]. Notably, *SIG5* is induced by high light, low temperature, high salinity, and osmotic stress, and specifically activates the blue-light-responsive *psbD* promoter to facilitate photosystem II (PSII) repair, thereby enhancing stress survival and recovery. This blue light/stress-responsive module was acquired early during land plant evolution and has been conserved, highlighting its fundamental importance for adaptation to fluctuating terrestrial environments [4,5]. *SIG6* activity is also modulated by phosphorylatable sites; changes in its phosphorylation state can reset promoter selectivity, providing a mechanistic basis for stress signals to impinge on PEP-dependent transcription [6], and it can further influence nuclear gene expression via plastid-to-nucleus retrograde signaling [7]. More broadly, because chloroplasts are highly sensitive to environmental stresses, σ factors—acting as a valve for PEP promoter choice—contribute to maintaining photosynthetic complex homeostasis, balancing ROS production and scavenging, and shaping stress phenotypes through retrograde signaling [8]. Collectively, σ factors represent a central hub linking chloroplast transcription initiation, developmental progression, and environmental signaling [9]. With advances in genomics and molecular technologies, systematic dissection of σ-factor family members and their regulatory networks will deepen our understanding of plastid transcriptional control and plant adaptive regulation, and may enable more precise molecular targeting for crop stress-resilience improvement.

With intensifying global warming and increasingly anomalous precipitation regimes, the frequency and magnitude of extreme climate events continue to rise, and drought has emerged as a major threat to food security. Climate change has been shown to broadly depress crop yields, with particularly pronounced impacts in low-latitude developing countries [10]. As the primary organ for sensing soil conditions and acquiring water and nutrients, roots play central roles in drought perception and adaptation. Under water-limited conditions, plants typically enhance drought adaptation through three major strategies. First, they extend roots into deeper or wider soil layers to access more stable water sources, manifested as deeper rooting, steeper (more vertical) root angles, longer primary roots, and increased deep lateral rooting [11], Second, they increase water uptake capacity by elevating root length density, expanding the surface area of roots (and root hairs), and adjusting the root-to-shoot ratio [12]. Third, they exploit high developmental plasticity—driven by hormonal regulation, hydraulic conductivity, and stress-responsive gene expression—to dynamically reprogram root branching patterns, growth direction, and spatial density [13]. Collectively, drought commonly induces fewer lateral roots, thicker root diameters, preferential allocation to deeper rooting, and a higher root-to-shoot biomass ratio, thereby supporting drought avoidance and drought tolerance by stabilizing plant water supply, alleviating shoot stress, and sustaining transpiration and photosynthetic activity.

The relationship between root development and stress tolerance has become a central topic in plant physiology and crop improvement. Beyond water and nutrient uptake and anchorage, processes underlying root growth—cell division, cell expansion, and root system architecture formation—profoundly influence the operation of antioxidant defense networks under stress. During root development, antioxidant enzymes and non-enzymatic components, particularly glutathione (GSH) within the ascorbate–glutathione (AsA–GSH) cycle, are critical for maintaining redox homeostasis in root cells and mitigating stress-induced damage. From a developmental perspective, following primary root emergence and elongation, lateral roots are initiated from pericycle cells adjacent to the vascular tissues, forming a branched root system. This process requires tight control of cellular activity and redox status. Notably, ascorbate peroxidase (APX), a key enzyme for hydrogen peroxide (H_2_O_2_) detoxification, has also been implicated in lateral root initiation and branching regulation [14]. Under abiotic stresses such as drought and salinity, roots are often the first to perceive water deficit or ionic stress, leading to the accumulation of reactive oxygen species (ROS), including superoxide and H_2_O_2_, which can damage membranes, proteins, and nucleic acids. In response, root antioxidant enzymes—superoxide dismutase (SOD), APX, catalase (CAT), and peroxidases (POD)—are rapidly activated to constrain ROS-induced injury. Meanwhile, non-enzymatic antioxidants such as GSH contribute to redox buffering via the interconversion between reduced GSH and oxidized glutathione (GSSG) within the AsA–GSH cycle, thereby helping maintain cellular redox balance [15]. Multiple root-focused studies support this linkage. In Medicago sativa (alfalfa), drought-tolerant genotypes display significantly higher CAT, POD, and SOD activities in roots than drought-sensitive genotypes, together with lower malondialdehyde (MDA; a lipid peroxidation product) levels, indicating better maintenance of membrane integrity and antioxidant capacity [16]. A meta-analysis of root stress responses further showed that mycorrhizal symbiosis significantly increases root SOD, CAT, POD, and APX activities under drought, concomitantly reducing H_2_O_2_ accumulation [17]. Overall, systematically integrating root developmental traits with stress-related biochemical indicators—such as SOD, APX, POD, CAT, GSH, and MDA as a damage marker—facilitates mechanistic understanding of how plants coordinately optimize root architecture and antioxidant defenses to improve stress adaptation [18]. From a breeding and agronomic perspective, selection for stress-resilient crops should therefore consider not only root morphological traits but also the responsiveness of root antioxidant enzyme activities and non-enzymatic antioxidants such as GSH. A deeper understanding of the root development–antioxidant defense nexus is essential for stabilizing crop yields under drought, salinity, heat, and other climate-related stresses.

Plant sigma factors have been mainly studied for their roles in plastid gene transcription and aboveground development, particularly in chloroplast biogenesis, leaf pigmentation, and photosynthetic establishment. However, whether sigma factors also contribute to belowground development, especially root system formation, remains largely unclear. In plants, shoots and roots are tightly integrated through long-distance transport of nutrients and signaling molecules. Increasing evidence has shown that alterations in shoot development, photosynthesis, or chloroplast function can be transmitted to the root through shoot-to-root communication, thereby modulating root growth and development [19]. For example, the mobile transcription factor HY5 coordinates shoot-derived light signaling with root growth and nutrient responses [20], while changes in chloroplast function may also influence root development through sugar signaling [21] and mobile RNAs [22]. These findings suggest that chloroplast-related genes may play important roles in coordinating shoot–root development. *ZmSIG2A*, an important member of the maize sigma factor family, is closely associated with chloroplast development, yet its role in root development remains unknown. In this study, taking advantage of the contrasting root phenotypes of two *ZmSIG2A* mutants, we investigated the root growth-promoting effect mediated by the upregulation of *zmsig2a^Val480del^* in *eal1-1* and explored its underlying molecular basis. By integrating phenotypic characterization, physiological analyses, and regulatory network investigation, this study aims to reveal the potential function of *ZmSIG2A* in maize root development and to provide new insight into the biological roles of sigma factors in stress adaptation.

## 2. Results

### 2.1. The Maize eal1-1 Mutant Exhibits Enhanced Root Growth and Improved Drought Resilience

**Figure 1:**
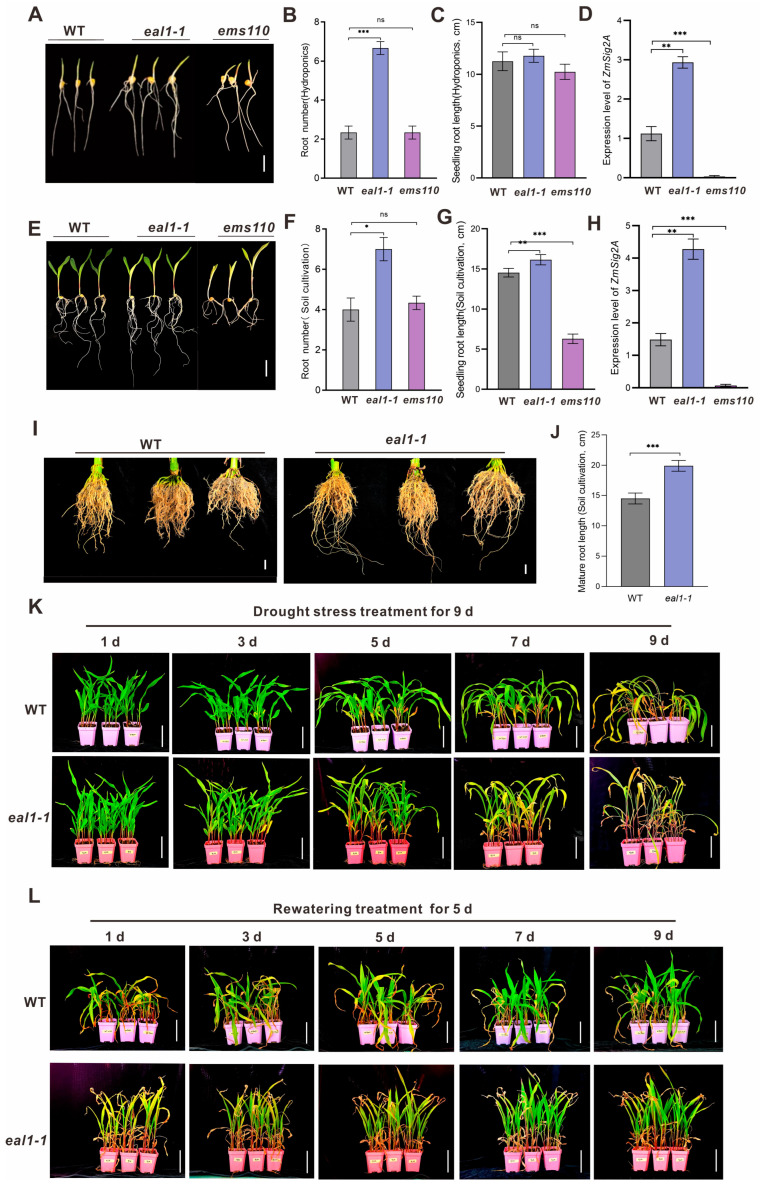
Root phenotypes and drought tolerance assessment of maize *ZmSIG2A* mutants. (**A**) Root morphology of WT, *eal1-1*, and *ems110* seedlings after 3 days of hydroponic culture. Scale bars = 2 cm. (**B**,**C**) Quantification of root number and root length corresponding to (**A**). (**D**) Relative expression of *ZmSIG2A* in hydroponically grown roots of *eal1-1* and *ems110*. (**E**) Root morphology of WT, *eal1-1*, and *ems110* seedlings after 6 days of soil cultivation. Scale bars = 1 cm. (**F**,**G**) Quantification of root number and root length corresponding to (**E**). (**H**) Relative expression of *ZmSIG2A* in soil-grown roots of *eal1-1* and *ems110*. (**I**) Root morphology of WT and *eal1-1* plants at the adult stage. Scale bars = 2 cm. (**J**) Quantification of adult root length corresponding to (**I**). (**K**) Drought treatment of 20-day-old WT and *eal1-1* plants at 28 °C for 9 consecutive days; representative phenotypes were photographed on days 1, 3, 5, 7, and 9. (**L**) Rewatering after drought stress; recovery phenotypes were monitored and documented daily from 1 to 5 d after rewatering. Scale bars = 10 cm. All data are presented as mean ± SD (n = 3). ns, not significant; * *p* < 0.05, ** *p* < 0.01, *** *p* < 0.001. Statistical significance was determined by one-way ANOVA followed by Tukey’s HSD multiple-comparisons test.

The maize *eal1-1* and *ems110* harbor different mutations in *ZmSIG2A*, resulting in divergent root phenotypes. *eal1-1* is a hypomorphic allele with a Val deletion in the σ4 domain (*zmsig2a^Val480del^*), whereas *ems110* carries a C>T mutation in exon 5 that causes premature termination and prevents plants from reaching maturity. At the seedling stage, *eal1-1* produced significantly more roots than WT under both 3 d hydroponic and 6 d soil-grown conditions (Figure 1A–C), while *ems110* showed no difference in root number. Root length was similar among genotypes in hydroponics, but in soil *eal1-1* had longer roots and *ems110* had shorter roots (Figure 1E–G). Adult-stage root measurements confirmed increased root length in *eal1-1* (Figure 1I,J), whereas *ems110* could not be evaluated due to lethality. We further examined the tissue expression pattern of *ZmSIG2A* in WT and found that it is expressed in roots, stems, and leaves (Figure S1A), with relatively higher expression in leaves. Notably, *ZmSIG2A* transcript levels were significantly elevated in the roots of *eal1-1* under both hydroponic and soil conditions (Figure 1D,H), suggesting that enhanced root growth may be associated with increased *zmsig2a^Val480del^* expression. Consistently, *OsSIG2A* overexpression in rice promotes primary root elongation and lateral root formation (Figure S1B,C), supporting a conserved root-promoting role of *SIG2A* factors in monocots. To evaluate whether enhanced root growth in *eal1-1* confers greater drought tolerance, we conducted a dehydration–rehydration assay comparing *eal1-1* and WT. *eal1-1* wilted more slowly than WT during the early phase of drought, but exhibited more severe chlorosis and senescence under prolonged stress, potentially due to impaired chloroplast development caused by the *ZmSIG2A* mutation. Importantly, after 5 d of rewatering, *eal1-1* recovered faster and showed superior post-stress growth relative to WT (Figure 1K,L), suggesting that its more developed root system facilitates water replenishment and promotes growth recovery following drought stress.

### 2.2. DAP-Seq Reveals That zmsig2a^Val480del^ Regulates Stress-Related Signaling Pathways

**Figure 2:**
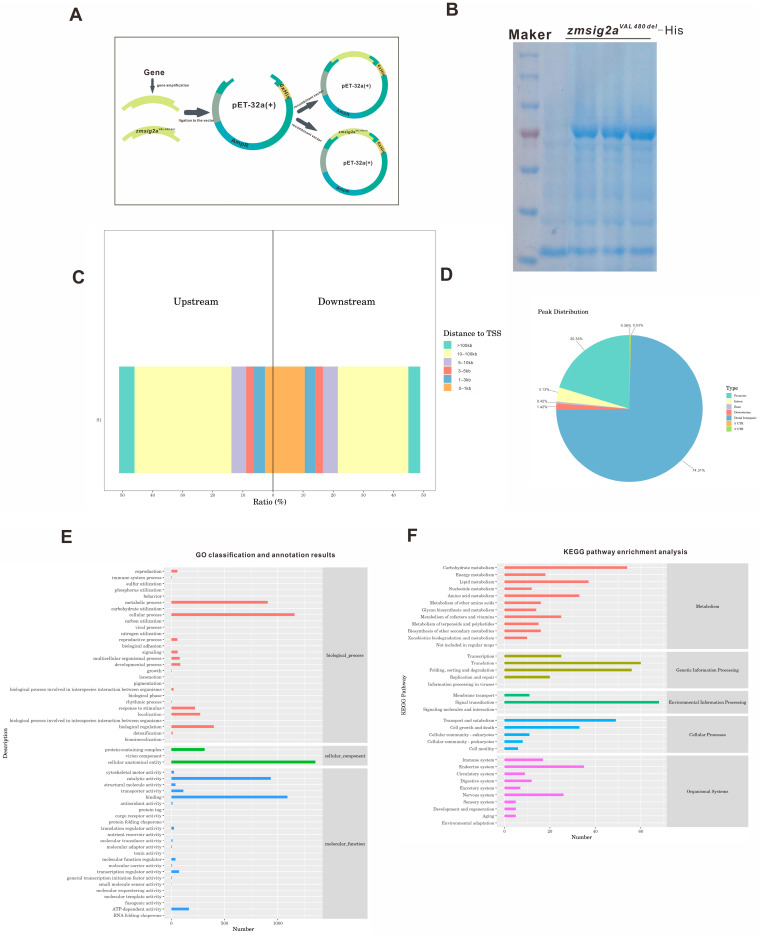
Genome-wide distribution and functional enrichment of DAP-seq binding sites for *zmsig2a^Val480del^*. (**A**) Schematic diagram of the recombinant construct for *zmsig2a^Val480del^* (prokaryotic expression vector: pET-32a). (**B**) SDS–PAGE analysis of the purified *zmsig2a^Val480del^* protein. (**C**) Distribution of peak distances relative to the transcription start site (TSS). The *x*-axis indicates the proportion of peaks within each distance bin, and the *y*-axis indicates samples; colors denote peaks located at different upstream/downstream intervals from the TSS. (**D**) Genomic annotation of DAP-seq peaks across functional elements (promoter, exon, intron, UTR, and distal intergenic regions), showing predominant enrichment in distal intergenic regions. (**E**,**F**) GO and KEGG classification of *zmsig2a^Val480del^* DAP-seq target genes. 3.4 LRR-RLK enrichment on Chr4 suggests *ZmSIG2A* may regulate root-related receptor kinases.

To examine the signaling pathways regulated by *zmsig2a^Val480del^*, we expressed and purified the recombinant protein in a prokaryotic system (Figure 2A,B) and performed DAP-seq to profile its genome-wide binding landscape. Peak–TSS analysis showed that binding sites occurred in both promoter-proximal and distal regions, with a substantial fraction located >10 kb from the nearest TSS (Figure 2C). Consistently, genomic annotation revealed strong enrichment in distal intergenic regions (74.31%), with only minor proportions in promoters (0.42%), exons (1.42%), introns (3.13%), and UTRs (Figure 2D), These results suggest that *zmsig2a^Val480del^* preferentially associates with distal cis-regulatory elements, potentially modulating target gene transcription via long-range regulatory mechanisms. Notably, integrated GO and KEGG analyses of *zmsig2a^Val480del^* DAP-seq target genes revealed a downstream regulatory network with dual attributes encompassing primary metabolism and stress responses (Figure 2E,F). At the GO level, target genes were broadly enriched in fundamental biological processes such as metabolic process, cellular process, and developmental process, while also showing significant enrichment in response to stimulus, immune system process, detoxification, and terms related to carbohydrate metabolism and the acquisition of key nutrients (C, N, P, and S). In the molecular function category, enrichments were mainly observed for catalytic activity, binding, transporter activity, molecular transducer activity, transcription regulator activity, and antioxidant activity.

Consistently, KEGG pathway classification showed that target genes were significantly enriched in core metabolic pathways, including carbohydrate metabolism, amino acid metabolism, and energy metabolism, as well as defense-associated pathways such as xenobiotics biodegradation and metabolism and biosynthesis of other secondary metabolites. In addition, a high proportion of targets were assigned to environmental information processing pathways, including signal transduction, signaling molecules and interaction, and membrane transport, and to categories closely linked to stress adaptation such as immune system, environmental adaptation, and cell growth and death. Collectively, these GO/KEGG annotations indicate that *zmsig2a^Val480del^* not only contributes to the regulation of maize growth, development, and metabolism, but also targets a suite of genes involved in signaling, membrane receptors, antioxidant capacity, and detoxification metabolism, thereby establishing a multilayered, interconnected regulatory network that likely underpins its role in maize adaptation to abiotic stress.

### 2.3. Enrichment of LRR-RLKs on Chr4 Suggests That zmsig2a^Val480del^ May Regulate Root-Associated Receptor Kinases

**Figure 3:**
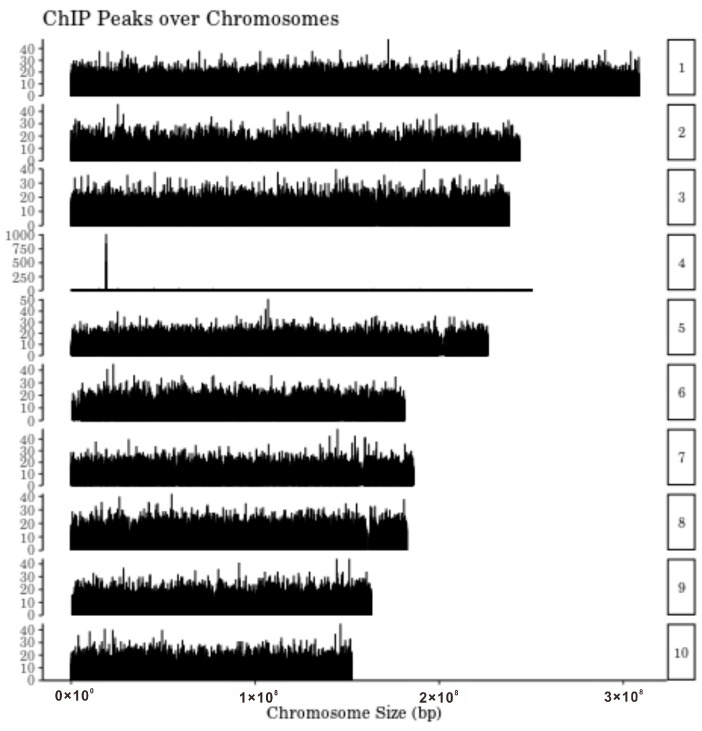
Genome-wide chromosomal distribution of DNA Affinity Purification sequencing (DAP-seq) peaks in *eal1-1* maize.

Genome-wide inspection of DAP-seq peak distributions (Figure 3) revealed varying degrees of enrichment across all maize chromosomes, indicating genomic regions potentially harboring transcription factor binding sites and regulatory hotspots. Notably, chromosome 4 (Chr4) displayed an exceptionally dense cluster of high-intensity peaks within specific intervals. To contextualize this pattern, we surveyed previously reported functional loci on Chr4 and found that this chromosome harbors multiple well-characterized QTLs and genes associated with key agronomic traits, including plant architecture, flowering time, stress tolerance, and kernel quality (Table 1). Given the root-promoting phenotype associated with *zmsig2a^Val480del^* perturbation, we focused on the receptor-like kinase (RLK) superfamily, particularly leucine-rich repeat RLKs (LRR-RLKs). A total of 205 LRR-RLKs have been annotated in the maize genome, with Chr4 containing the largest number (29 genes). Previous studies have shown that LRR-RLKs broadly participate in root system establishment and remodeling across plant species. They can sense soil-derived cues such as water and nutrient status and mechanical impedance to regulate primary root elongation and lateral root formation, thereby shaping root architecture and absorptive capacity. In addition, many LRR-RLKs influence root apical meristem activity, cell proliferation, and cortical patterning, underpinning root developmental plasticity and stress-induced root reprogramming. Therefore, LRR-RLKs are widely regarded as signaling hubs linking environmental perception to root developmental outputs.

In our DAP-seq dataset, the Chr4 peak-clustered region co-localized with 16 LRR-RLK family members (Table S1). Considering the transcriptional regulatory potential of *ZmSIG2A* and the central roles of LRR-RLKs in root development and hormone-signal integration, we propose that *ZmSIG2A*/*zmsig2a^Val480del^* may directly or indirectly regulate the expression of these LRR-RLK genes. This regulation may modulate root sensitivity to external stress cues by changing the abundance or composition of receptors at the plasma membrane, and it may also reshape root growth and developmental programs by affecting LRR-RLK–mediated peptide and phytohormone signaling.

**Figure 4:**
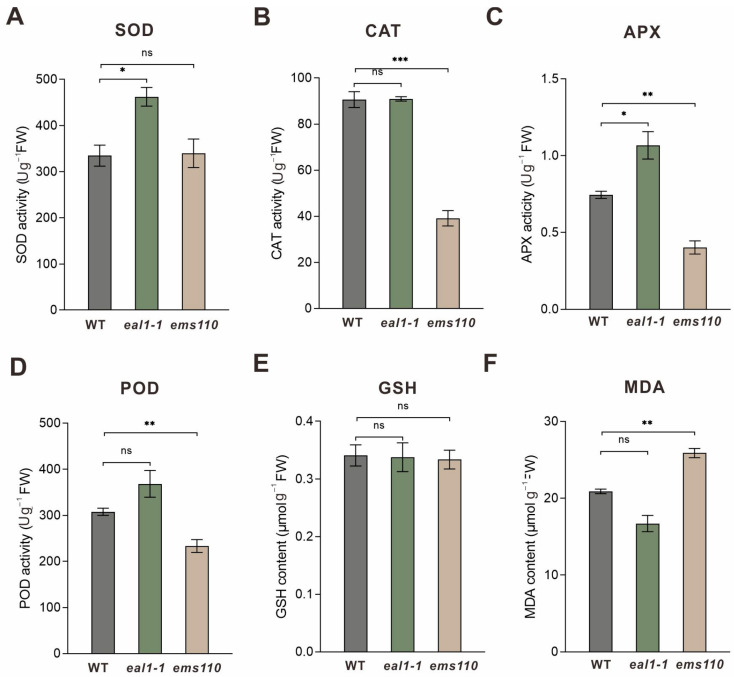
Activities of SOD, CAT, APX, and POD, and the contents of GSH and MDA in the roots of *eal1-1* and *ems110* maize seedlings. (**A**–**F**) Enzyme activity assays and metabolite measurements in roots of 13-day-old *eal1-1* and *ems110* seedlings. Root samples were collected at 13 days after sowing (DAS) for quantification of SOD, CAT, APX, and POD activities, as well as GSH and MDA levels. Data are presented as mean ± SD (n = 3). ns, not significant; * *p* < 0.05, ** *p* < 0.01, *** *p* < 0.001. Statistical significance was determined by one-way ANOVA followed by Tukey’s HSD multiple-comparisons test.

To further compare stress-associated enzymatic activities between the maize mutants *eal1-1* and *ems110*, we quantified SOD, POD, APX, CAT, and GSH levels, as well as MDA content in seedling roots. *eal1-1* exhibited significantly increased root biomass, with coordinated increases in SOD, POD, and APX activities, little change in CAT, no significant difference in GSH, and a slight decrease in MDA (Figure 4A–F). This pattern indicates an overall enhancement of ROS-scavenging flux: superoxide is efficiently dismutated to H_2_O_2_ by SOD, and H_2_O_2_ is subsequently removed primarily via the APX/POD branches. Although CAT activity remained unchanged, the net oxidative burden was reduced, leading to lower lipid peroxidation as reflected by decreased MDA. In addition, a more developed root system may further improve water uptake and osmotic adjustment capacity, thereby buffering stress impacts and supporting higher overall tolerance. In contrast, *ems110* showed decreased SOD, POD, and APX activities together with a marked reduction in CAT; GSH remained unchanged, whereas MDA increased significantly. These results suggest a concerted weakening of multiple enzymatic detoxification routes, likely promoting H_2_O_2_ accumulation and exacerbating membrane damage, consistent with reduced stress tolerance. Collectively, the comparison indicates that *eal1-1* maintains a more robust ROS homeostasis and lower oxidative injury, whereas *ems110* suffers from a ROS-scavenging bottleneck and aggravated membrane damage.

### 2.4. ZmMed31-Mediated Positive Regulation of ZmSIG2A Expression

**Figure 5:**
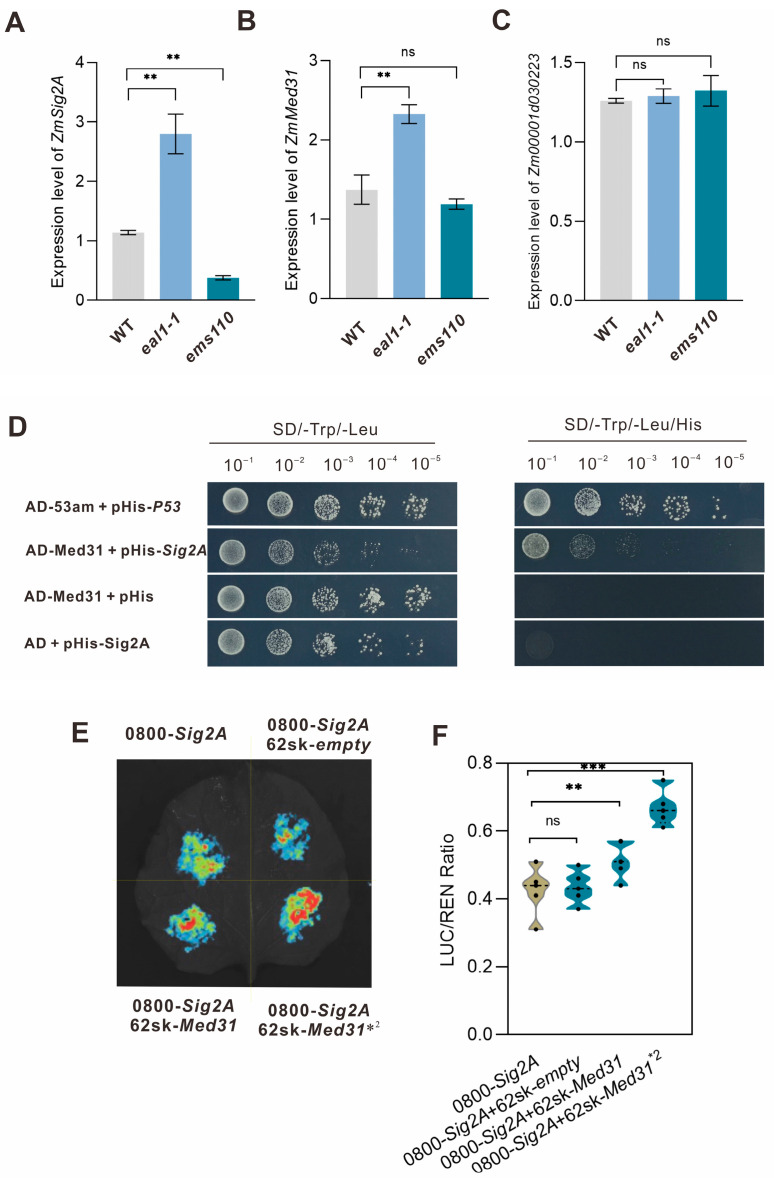
Expression analysis of candidate genes in *eal1-1* and *ems110* and validation of molecular interactions. (**A**–**C**) Differential expression of *ZmSIG2A*, *Zm00001d031902* (*ZmMed31*), and *Zm00001d030223* in *eal1-1* and *ems110*. Data are presented as mean ± SD (n = 3). ns indicate nonsignificant difference, ** *p* < 0.01 by two-sided Student’s *t*-test. (**D**) Yeast one-hybrid (Y1H) assay showing binding of *ZmMed31* to the *ZmSIG2A* promoter. (**E**,**F**) Dual-luciferase reporter assays in *Nicotiana benthamiana* leaves. Quantification of the LUC/REN ratio indicates that *ZmMed31* positively regulates the activity of the *ZmSIG2A* promoter fragment, *2 indicates ZmMED31 at a twofold bacterial suspension concentration. Images are representative of at least three independent biological replicates. Data are presented as mean ± SD (n = 3). ns indicate nonsignificant difference, ** *p* < 0.01, *** *p* < 0.001 by two-sided Student’s *t*-test.

To determine whether *ZmSIG2A* is regulated by stress-associated upstream factors, we performed a yeast one-hybrid (Y1H) screen using the pAbAi system to identify transcriptional regulators binding the *ZmSIG2A* promoter. After optimizing conditions, the minimal aureobasidin A (AbA) concentration that effectively suppressed bait autoactivation was determined and used for large-scale cDNA library screening. Positive colonies were subjected to PCR and sequencing (Figure S2B), yielding candidate interactors. Functional annotation indicated that these candidates are mainly involved in transcriptional regulation, signal transduction, and stress responses (Table 2), suggesting that *ZmSIG2A* expression is closely linked to endogenous regulatory networks and environmental cues. Among the hits, *Zm00001d031902* (*ZmMed31*) is associated with ABA signaling, implying ABA-dependent regulation of *ZmSIG2A*; *Zm00001d030223*, annotated as related to root development and cell cycle control, suggested potential integration with developmental status. We next examined transcript abundance in roots of *eal1-1* and *ems110*. *ZmMed31* and *ZmSIG2A* were both significantly upregulated in *eal1-1*, whereas *ZmSIG2A* was strongly downregulated in *ems110* with no significant change in *ZmMed31* relative to WT (Figure 5A,B). *Zm00001d030223* showed no significant differences in either mutant (Figure 5C). These data nominate *ZmMed31* as a primary upstream candidate. Using a pHIS2-based Y1H assays using the pHis2 system indicated that *ZmMed31* might specifically bind to the *ZmSIG2A* promoter (Figure 5D), hinting at its potential role in transcriptional regulation. Furthermore, dual-luciferase assays provide evidence that *ZmMed31* positively regulates *ZmSIG2A* transcriptional activity (Figure 5E,F). In light of previous reports that Mediator subunits participate in ABA signaling to regulate root development in *Arabidopsis*, and considering the root developmental phenotype observed in maize *eal1-1*, we hypothesize that *ZmMed31* could influence root system architecture in *eal1-1* by mediating ABA-related signaling and regulating *ZmSIG2A* transcription.

## 3. Discussion

### 3.1. ZmSIG2A Regulates Root Development and Drought Recovery in Maize

Global analyses of major staple crops (maize, rice, soybean, and wheat) show that from 1983 to 2009, drought reduced yields across 75% of harvested areas, causing an estimated US$166 billion in cumulative losses [27]. Climate change—via warming, increased evapotranspiration, and altered precipitation regimes—has further increased drought frequency and unpredictability [28]. Because drought tolerance is shaped by complex gene–environment interactions, conventional breeding has delivered only modest gains (2–4%) in major crops [29], Thus, dissecting drought-induced physiological, biochemical, and molecular responses, and integrating these insights into molecular breeding and precision agriculture, is essential for improving drought resilience. Here, we compared two maize *ZmSIG2A* mutant lines, *eal1-1* and *ems110*, which exhibited contrasting root phenotypes. At the seedling stage, *eal1-1* developed significantly more roots under both hydroponic and soil-grown conditions and showed increased root length specifically in soil, whereas *ems110* displayed WT-like root number but reduced root length in soil (Figure 1A–F). *zmsig2a^Val480del^* was significantly upregulated in *eal1-1*, which may promote root branching and elongation; Notably, root length differences in *eal1-1* were not obvious under hydroponic conditions but were significant under soil culture at both seedling and mature stages, indicating that this phenotype may depend on soil-related environmental factors. These results suggest that *ZmSIG2A* may participate in the plastic response of root growth to external environmental changes. It is noteworthy that *OsSIG2A*/*OsSIG2B* in rice show high homology with *ZmSIG2A*/*ZmSIG2B* in maize, indicating that *SIG2* genes are highly conserved among monocot species. Previous studies have shown that SIG2-type σ factors play important roles in plastid gene transcription and chloroplast development [30]. The high conservation of *SIG2* homologs between maize and rice therefore suggests functional similarity. In our cross-species validation experiments, overexpression of *OsSIG2A* in rice significantly promoted primary root elongation and increased lateral root number, providing evidence that *SIG2A* genes are involved in root developmental regulation in monocots and further supporting functional conservation across species. Given the high sequence similarity between maize and rice homologs, it is reasonable to speculate that *SIG2A* genes may jointly contribute to monocot root architecture by regulating chloroplast development and related retrograde signaling, energy metabolism, or hormone-responsive pathways.

In drought assays, *eal1-1* wilted more slowly than WT early during stress and recovered more rapidly after rewatering, with improved post-stress growth (Figure 1K,L), indicating that enhanced root development benefits rehydration and recovery, likely through improved water uptake and osmotic buffering. However, *eal1-1* exhibited stronger chlorosis and senescence under prolonged drought, indicating stage-dependent performance that may reflect impaired chloroplast development caused by the *ZmSIG2A* mutation. Together, these results suggest that *ZmSIG2A* is a potential regulatory node linking root development, drought recovery capacity, and chloroplast-associated metabolic integrity, In *eal1-1*, *ZmSIG2A* is strongly upregulated alongside increased root number and greater root length in soil, implying roles in root development and environmental responsiveness. Enhanced branching and elongation likely expand soil exploration and absorbing surface area, slowing shoot water potential decline and explaining the slower wilting and faster recovery after rewatering. By contrast, *eal1-1* shows stronger chlorosis and senescence under prolonged drought, indicating a stage-dependent trade-off, a larger root system benefits early drought resistance and recovery, but sustained stress is associated with impaired chloroplast function, reduced photosynthesis, and accelerated senescence. Thus, *ZmSIG2A* may sit at a nexus of chloroplast/metabolic status–source–sink allocation–root system construction, revealing a key balance between root water acquisition and leaf metabolic/photosynthetic stability.

### 3.2. ZmSIG2A Modulates LRR-RLKs and ROS Dynamics to Enhance Root Growth and Stress Tolerance in Maize

To test whether *zmsig2a^Val480del^* contributes to stress regulation in *eal1-1*, we used DAP-seq to profile its genome-wide binding and downstream targets. Target genes were significantly enriched in pathways associated with primary metabolism, membrane transport, signal transduction, and antioxidant processes (Figure 2E,F), indicating that *zmsig2a^Val480del^* may coordinate a regulatory program linking growth-related metabolism with stress responses. Notably, DAP-seq peaks showed pronounced clustering on Chr4 and overlapped with multiple LRR-RLK loci, providing testable candidates potentially underlying the enhanced root phenotype. As key hubs that couple environmental perception to growth reprogramming, LRR-RLKs integrate water status, peptide cues, and phytohormone signaling to regulate root apical meristem activity, lateral root formation, and tissue patterning [31,32,33], These observations support a putative *ZmSIG2A*–LRR-RLK module that may promote root enhancement in *eal1-1* by tuning receptor abundance or composition and thereby altering sensitivity to environmental and hormonal inputs. Targeted regulation of the LRR-RLK gene cluster by *ZmSIG2A* may integrate external cues with endogenous signaling, shifting meristem activity, lateral root formation, and tissue development, and thereby driving the enhanced root proliferation and soil-dependent elongation of *eal1-1*. Physiologically, contrasting ROS homeostasis helps explain the divergence between *eal1-1* and *ems110*. Because receptor kinase pathways often modulate ROS dynamics and root development is highly ROS-sensitive, the coordinated upregulation of SOD/POD/APX together with reduced MDA in *eal1-1* (Figure 4A–F) suggests stronger antioxidant buffering that limits chronic oxidative damage while preserving local ROS signals for cell division and lateral root initiation. By contrast, *ems110* shows broadly reduced antioxidant enzyme activity—especially CAT—and elevated MDA, indicating impaired ROS detoxification and increased membrane injury. Taken together, the *ZmSIG2A*–LRR-RLK module may constitute a key pathway in eal1-1 that connects signal perception to phenotypic regulation: it converts environmental information into root developmental outputs and promotes rapid adaptation through the maintenance of ROS homeostasis.

### 3.3. Mediator Subunit ZmMed31 Regulates ZmSIG2A to Coordinate Root Morphogenesis and Stress Resilience in Maize

Y1H screening results indicate that the *ZmSIG2A* promoter is recognized by multiple candidate factors involved in transcriptional regulation, signal transduction, and stress responses, suggesting that *ZmSIG2A* may act as a key integrator of various signaling pathways. *ZmMed31*, as a Mediator subunit, is known to interact with specific transcription factors to regulate downstream gene expression. For example, *AtMED25*, another Mediator subunit, modulates hormone-responsive gene expression by selectively interacting with transcription factors in different hormonal pathways. In the jasmonate (JA) pathway [34], MED25 interacts with MYC2 to positively regulate its target genes, while in the abscisic acid (ABA) pathway, MED25 associates with ABI5 to inhibit its target gene expression. These findings underscore Mediator’s multifaceted role in hormone signaling and transcriptional regulation. Additionally, *AtMED31* has been shown to influence root development through the SHR–SCR pathway in Arabidopsis [35]. In *eal1-1*, we observed that the expression of *ZmMed31* and *zmsig2a^Val480del^* are upregulated in parallel, which was further confirmed by yeast one-hybrid and dual-luciferase assays showing that *ZmMed31* directly binds to the *ZmSIG2A* promoter and positively regulates its transcription. This suggests that *ZmMed31* functions as an upstream regulator of *ZmSIG2A*. We hypothesize that the elevated expression of *ZmMed31* in *eal1-1* may enhance the transcriptional regulation of hormone-related genes, potentially contributing to improved stress tolerance. Moreover, *ZmMed31* may bridge hormone signaling with the transcriptional regulation of *ZmSIG2A*, influencing downstream signaling networks and root antioxidant systems. Based on phenotypic, physiological, DAP-seq, and molecular interaction data, we propose that in *eal1-1*, *ZmMed31*, through its interaction with hormone signaling transcription factors, regulates the transcription of *zmsig2a^Val480del^*. This, in turn, may modulate downstream pathways, including those involving LRR-RLK receptor kinases, transporters, and antioxidant/detoxification genes, which are crucial for root development and ROS homeostasis, thereby enhancing stress adaptation. Importantly, this regulatory process is unlikely to be restricted to local root responses, but may instead involve systemic shoot–root communication. Because leaves are the major sites of photosynthesis and environmental sensing, changes in leaf developmental status, photosynthetic efficiency, and hormone or metabolite levels can be transmitted to roots through long-distance signaling, thereby influencing root growth and physiology. As a chloroplast development-related factor, transcriptional changes in *ZmSIG2A* may therefore affect not only chloroplast function and metabolic status in shoots, but also root growth and antioxidant defense through hormone signaling, sugar signaling, or other shoot-derived signals. In this context, the *ZmMed31*–*ZmSIG2A* module in *eal1-1* may function as a bridge linking shoot chloroplast status to root stress responses, thereby coordinating whole-plant development and environmental adaptation. Future studies will aim to further investigate *ZmSIG2A*’s direct binding and regulatory effects on LRR-RLK promoters through DAP-qPCR, EMSA, and dual-luciferase assays. Additionally, generating single and double mutants of key LRR-RLK genes on Ch4, along with *ZmSIG2A* overexpression constructs, will allow us to dissect the functional roles and interactions of these kinases in the *ZmSIG2A*-mediated pathway. Ultimately, integrating key genetic loci with natural variations in maize breeding programs could provide valuable molecular targets for developing maize varieties with improved root architecture, nutrient efficiency, and stress resilience.

## 4. Materials and Methods

### 4.1. Plant Materials and Growth Conditions

Maize (*Zea mays* L.) inbred line B73 was used as the wild type. The mutant trait of *eal1* (etiolated/albino leaf 1) [36] was backcrossed to inbred line B73 for 5 generations to construct a near-isogenic line *eal1-1* (B73*^eal1eal1^*). The *ems110* mutant (B73 background) was obtained from an EMS-mutagenized seed library via the MEMD database (http://www.elabcaas.cn/memd/) (accessed on 11 June 2024). *OsSIG2A*(*Os11g0448400*) overexpression lines were generated in the rice (*Oryza sativa*) cultivar ZH11 background.

### 4.2. Plant Growth Conditions

Maize seedlings were primarily grown in a greenhouse under a 16 h light/8 h dark photoperiod, with day/night temperatures of 28 °C/21 °C and 70% relative humidity. Seeds of *eal1-1*, WT, and *ems110* were sown in 9 cm-diameter pots filled with a soil–vermiculite mixture and watered every 3 days under normal conditions. At 13 d after sowing, roots from some seedlings were harvested for enzyme activity assays. Each treatment included three biological replicates, each consisting of pooled root samples from five seedlings. Each biological replicate was analyzed with three technical replicates, and the mean value was used for statistical analysis. The remaining plants were transferred to the field and grown to maturity for root phenotyping. For hydroponic culture, seeds of *eal1-1*, WT, and *ems110* were sterilized, germinated at 28 °C for 2 d, transferred to germination paper, and then grown hydroponically in greenhouse tanks. Drought tolerance assays of *eal1-1* and WT were conducted in a 28 °C greenhouse. Seedlings were subjected to 9 d of water deprivation followed by 5 d of rewatering, and their phenotypes were then recorded. Because *ems110* plants usually died at around 20 d after germination, they were not included in the drought assay. Rice seeds were germinated in a 28 °C greenhouse, then transplanted into soil and grown in the field to maturity for phenotypic imaging.

### 4.3. RNA Extraction and Gene Expression Analysis

The roots of *eal1-1*, WT, and *ems110* plants were collected at the rapid growth stage (13 days after sowing)with three biological replicates. Total RNA was extracted using TRIzol Reagent (Takara, Japan), treated with DNase I, and reverse-transcribed into cDNA. RT–qPCR was performed on a CFX Connect Real-Time PCR System using SYBR Premix Ex Taq (Takara, Japan). The RT-qPCR thermal cycling conditions were as follows: an initial denaturation at 94 °C for 30 s, followed by 40 cycles consisting of denaturation at 94 °C for 5 s, and annealing/extension at 60 °C for 30 s. A final melting curve analysis was performed (94 °C for 15 s, 60 °C for 1 min, followed by continuous heating to 94 °C with fluorescence measurements) to confirm the specificity of the amplicons. The *ZmActin* gene (*Zm00001d010159*) served as the internal reference, and relative transcript levels of *ZmSIG2A* (*Zm00001eb169360*) and other root development-related genes were calculated. Gene expression levels were calculated using the ΔCT method in Microsoft Excel [37]. Primer sequences are provided in Table S2.

### 4.4. Prokaryotic Expression and Purification of Recombinant Protein

The coding sequences (CDSs) of *zmsig2a^Val480del^* were cloned into the pET-32a vector, and confirmed recombinant plasmids were transformed into *E. coli* BL21. Transformants were cultured at 37 °C, and expression of N-terminal His-tagged recombinant proteins was induced at 16 °C with 0.6mM isopropyl β-D-1-thiogalactopyranoside (IPTG) for 4–10 h. His-tagged proteins were purified by Ni–NTA affinity chromatography using Ni–NTA agarose (Invitrogen, Carlsbad, CA, USA). The purified proteins were concentrated by ultrafiltration, desalted and buffer-exchanged by dialysis, then mixed 1:1 with pre-chilled glycerol and stored for subsequent molecular assays. 60 μL of protein was mixed with 15 μL of SDS loading buffer and denatured at 98 °C for 12 min. The denatured protein was then loaded onto a 12–20% gradient SDS-polyacrylamide gel for analysis of protein purification success [38].

### 4.5. DNA Affinity Purification Sequencing (DAP-Seq)

DAP-seq combines in vitro protein expression with high-throughput sequencing to map transcription factor binding sites genome-wide [39]. Roots from 14-day-old *eal1-1* seedlings grown at 28 °C were collected as experimental samples. For each replicate, roots from 15 seedlings were pooled, with three independent biological replicates in total. Genomic DNA was extracted and incubated with purified *zmsig2a^Val480del^* protein for DNA–protein binding. The specifically bound DNA fragments were submitted to BioMarker (Beijing, China) for sequencing on the Illumina NovaSeq platform. After quality filtering, reads were aligned to the maize reference genome B73 RefGen_v5, and downstream analyses were performed using DESeq2.

### 4.6. Yeast One-Hybrid Library Screening

A fragment of the *ZmSIG2A* promoter was amplified from maize genomic DNA using Primer5.0 designed primers containing pAbAi homologous arms, with TOYOBO KOD FX high-fidelity polymerase. The PCR product was recombined into pBait-pAbAi to generate pro-*ZmSIG2A*-pAbAi, which was transformed into Y1HGold and selected on SD/-Ura. Autoactivation was assessed on increasing aureobasidin A (AbA) concentrations to determine the minimum inhibitory concentration (MIC). Competent bait yeast cells were then co-transformed with a maize Root cDNA library, and transformants were selected on SD/-Leu + AbA plates at 30 °C for 12–24 h. Positive colonies were verified by colony PCR using T7-F/LD-R, followed by Sanger sequencing and NCBI BLAST identification. Confirmed positives were preserved for subsequent validation [40].

### 4.7. Yeast One-Hybrid (Y1H) Validation

The coding sequences of *ZmMed31*(*Zm00001d031902*) and *Zm00001d030223* were cloned into pGADT7, and the *ZmSIG2A* promoter was cloned into pHIS2. The resulting constructs were co-transformed into yeast strain AH109, and transformants were selected on SD/-Leu/-Trp medium. Positive colonies were then plated on SD/-Leu/-Trp/-His medium; growth on this medium indicated promoter binding and activation of the HIS3 reporter, conferring histidine prototrophy.

### 4.8. Dual-Luciferase Reporter Assay

A dual-luciferase assay was performed to evaluate transcriptional regulation of the *ZmSIG2A* promoter [41]. The *ZmSIG2A* promoter was cloned into pGreenII 0800-LUC to generate the reporter construct, and the *ZmMed31* coding sequence (CDS) was inserted into pGreenII 62-SK under the CaMV 35S promoter to generate the effector. After sequence verification, both constructs were introduced into *Agrobacterium tumefaciens* strain GV3101 and co-infiltrated into *Nicotiana benthamiana* leaves using a needleless syringe (bacterial suspension OD600 = 0.5–1.0). Plants were maintained at 25 °C under high humidity, and leaves were harvested 48 h post-infiltration for luciferase measurement using an in vivo imaging system. D-luciferin potassium salt was applied to the abaxial leaf surface prior to signal detection. Empty-vector combinations (pGreenII 0800-LUC and pGreenII 62-SK) served as negative controls. Firefly luciferase(LUC) and Renilla luciferase (REN) activities were measured using a Dual-Luciferase Reporter Assay Kit (Vazyme, Nanjing, China; DL101-01). The relative LUC activity was represented by the ratio of LUC to REN luminescence. Experiments were performed in three independent replicates, and data are presented as mean ± SE.

### 4.9. Biochemical Measurements

Root tissues were collected from *eal1-1*, WT, and *ems110* maize seedlings at 13 days after sowing. Approximately 0.5 g of fresh root tissue was harvested, immediately frozen in liquid nitrogen, and stored at −80 °C until analysis. The activities or contents of SOD (BC0170), CAT (BC0205), APX (BC0220), POD (BC0090), GSH (BC1175), and MDA (BC0025) were determined using commercial assay kits from Solarbio (Beijing, China) [42]. For extraction, the samples were homogenized in a precooled mortar with 1 mL of the corresponding extraction buffer under ice-cold conditions, followed by centrifugation at 12,000 rpm for 10 min at 4 °C. The supernatants were collected for subsequent assays. All procedures were performed according to the manufacturer’s instructions, and absorbance was measured at the specified wavelengths using a GENESYS 30 visible spectrophotometer (Thermo Fisher Scientific, Waltham, MA, USA). The concentrations or enzyme activities were calculated based on the corresponding standard curves.

## 5. Conclusions

Our study investigated *ZmSIG2A* in maize root development, drought recovery, and ROS homeostasis. We found that the upregulation of *zmsig2a^Val480del^* in the *eal1-1* mutant may promote root development and enhance water uptake, thereby improving drought recovery. DAP-seq analysis identified *ZmSIG2A* target genes enriched in LRR-RLK and related signaling pathways, suggesting its involvement in the coordinated regulation of root growth and stress responses. Additionally, we identified *ZmMed31* as an upstream regulator of *ZmSIG2A*, potentially linking hormone signaling transcription factors to *ZmSIG2A* transcriptional regulation, which in turn affects root antioxidant systems and downstream signaling outputs. Overall, this study provides new evidence and insights into the potential regulatory roles of *ZmSIG2A* in root development, drought recovery, and ROS homeostasis, contributing to our understanding of maize Sigma factors in stress response regulation.

## Figures and Tables

**Table 1:** Summary of functional annotations for reported genes on maize Chr4.

Number	Chr	GeneID	Function Description
1	4	LRR-RLK	Receptor kinases participate in signal transduction [23]
2	4	ZmOCL5	Major-effect QTL for Leaf Rolling Index [24]
3	4	Zm00001d052910	Major-effect QTL for kernel number per row [25]
4	4	ZmCLA4	Regulation of the leaf angle (LA) trait [26]
5	4	Vgt1	Regulation of maize flowering time (tasseling stage)
6	4	KRN2	Regulation of ear row number in maize
7	4	ZmCCT9	Regulation of Photoperiod Sensitivity and Flowering Time in Maize
8	4	glossy4 (gl4)	Involved in the biosynthesis of cuticular wax alkanes
8	4	amylose extender1	Encoding the starch branching enzyme SBEIIb
9	4	sh2 (shrunken2)	Encoding the large subunit of ADP-glucose pyrophosphorylase

**Table 2:** Functional annotation results of yeast colony sequences based on Blast alignment.

Number	Gene ID	Function Description
1	*Zm00001d031902*	Mediator of RNA polymerase II transcription subunit 31
2	*Zm00001d030223*	ATP binding protein
3	*Zm00001d026386*	2-methoxy-6-polyprenyl-1,4-benzoquinol methylase, mitochondrial
4	*Zm00001d010368*	derlin1-1 derlin1-1 sor protein
5	*Zm00001d012161*	60S ribosomal protein L5-1
6	*Zm00001d049496*	MOB kinase activator-like 1B

## Data Availability

All datasets generated for this study are included in the article/Supplementary File.

## Supplementary Materials

The following supporting information can be downloaded at: https://www.mdpi.com/article/10.3390/plants15071057/s1. Figure S1. Tissue-specific expression of *ZmSIG2A* in maize and phenotypic characterization of *OsSIG2A*-overexpressing rice lines. Figure S2. Determination of the minimal aureobasidin A (AbA) concentration for yeast one-hybrid library screening and PCR identification of interacting colonies. Table S1. DAP-identified LRR-RLK target genes on maize Chr4. Table S2. Primers used in this study.
