# Supplementary material for: ZmMed31–ZmSIG2A Coordinates ROS Homeostasis and LRR-RLK Signaling to Regulate Root Development
Source: Plants (Basel). 2026 Mar 30;15(7):1057. doi: 10.3390/plants15071057 (PMC13074371; doi:10.3390/plants15071057)
Supplement: Supplementary file 1 [file plants-15-01057-s001.zip › Supplementary files.pdf]

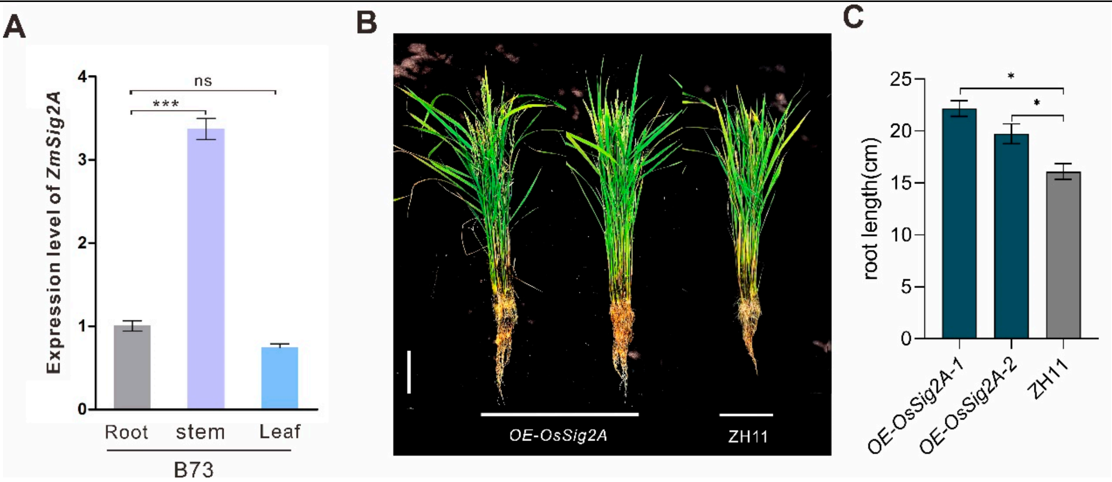

Figure S1 Tissue-specific expression of *ZmSIG2A* in maize and phenotypic characterization of *OsSIG2A*-overexpressing rice lines

（A）Differential expression of *ZmSIG2A* in roots, stems, and leaves of maize B73.（B） Root phenotypes of *OE-OsSIG2A* and ZH11 rice plants. (C) Quantification of root length. Data are presented as mean ± SD (n = 3). ns indicate nonsignificant difference, *$p < 0.05$, ***$p < 0.001$; Student’s t-test.

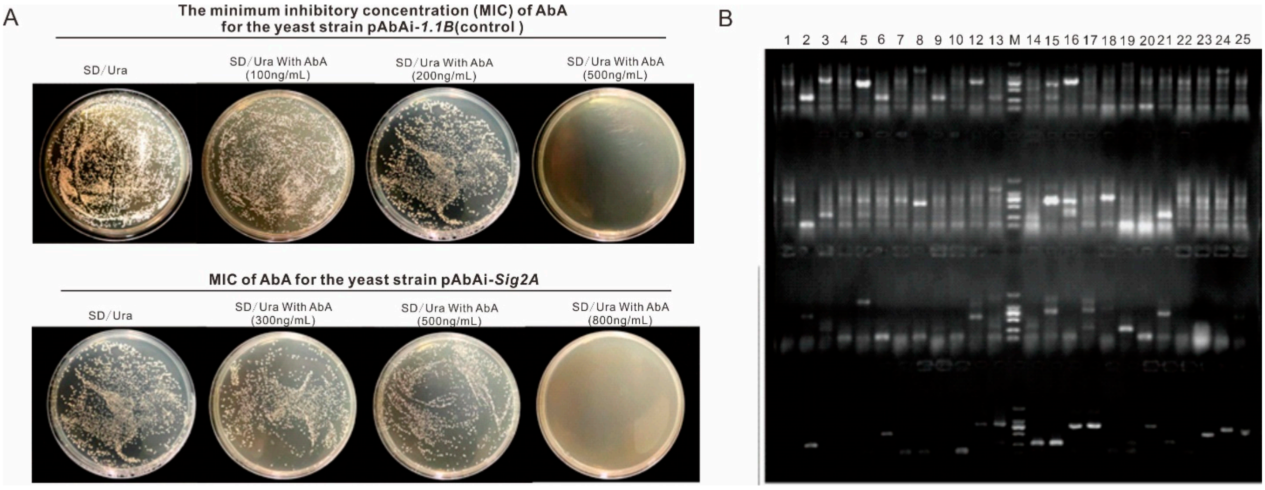

Figure S2. Determination of the minimal aureobasidin A (AbA) concentration for yeast one-hybrid library screening and PCR identification of interacting colonies.

(A) Determination of the minimal inhibitory concentration of AbA for pAbAi-*Sig2A* yeast strains; pAbAi-*1.1B* was used as a control strain with a known inhibitory concentration. (B) Agarose gel electrophoresis results of colony PCR from yeast transformants.

Table S1 DAP-identified LRR-RLK target genes on maize Chr4

编号	Chr	**Locus**	基因 **ID**
1	4	CDS	Zm00001eb184720
2	4	CDS	Zm00001eb177640
3	4	Promoter (0-1kb)	Zm00001eb196950
4	4	Promoter (0-1kb)	Zm00001eb174320
5	4	CDS	Zm00001eb174570
6	4	CDS	Zm00001eb200690
7	4	CDS	Zm00001eb184650
8	4	CDS	Zm00001eb184750
9	4	CDS	Zm00001eb184670
10	4	CDS	Zm00001eb184730
11	4	CDS	Zm00001eb179770
12	4	CDS	Zm00001eb204660
13	4	CDS	Zm00001eb184760
14	4	CDS	Zm00001eb207890
15	4	Promoter (1-2kb)	Zm00001eb187350
16	4	CDS	Zm00001eb165960

Table S2. Primers used in this study

Primer Name	Forward (5'–3')	Reverse (5'–3')	Application
ZmSig2A	atggcgtgcctggcgccg	tcaccaattgcctactggcacca	Cloning *ZmSIG2A* sequence
OsSig2A	atggcgtgcctcgcgccgca	tcaccaattgcctactggtacca	Cloning OsSIG2A sequence
qZmSIG2A	cgccgcaaagaagagagcta	cggtagttcgtagtaggccg	qRT-PCR
qZmMed31	gtagctccacctgttcctgc	agagtctcaatcatctttcttcctt	
Zm00001d030223	ttcgccatcgccgacttttc	tcactttgcaaacggctcac	
Med31-AD	aacatggaggccag-tgaattcatgtccgacgtcga-gaaccc	cagctcgagctcgatggatcctag-cacagacaaaatagcaagga	Y1H
Sig2A-p-phis	gactcac-tatagggcgaattcgcctcgcctgctaacaaaca	cggatcgattcgcgaacgcgtcggg-gagccagggctggc	
Sig2A- p2-0800	ctatagggcgaattgggtac-cgaactcaagggtcacgg	atctccac-cgcggtggcggccgccccgcc-tacacgaaccaaaa	Dual-Luciferase Reporter Assay
ZmMed31-62sk	agctccaccgcggtggcggccg-catgtccgacgtcgagaaccc	tcagcgtaccgaattggtacc tag-cacagacaaaatagcaagga	
