# Supplementary figures and images for: ZmMed31–ZmSIG2A Coordinates ROS Homeostasis and LRR-RLK Signaling to Regulate Root Development
Source: Plants (Basel). 2026 Mar 30;15(7):1057. doi: 10.3390/plants15071057 (PMC13074371; doi:10.3390/plants15071057)

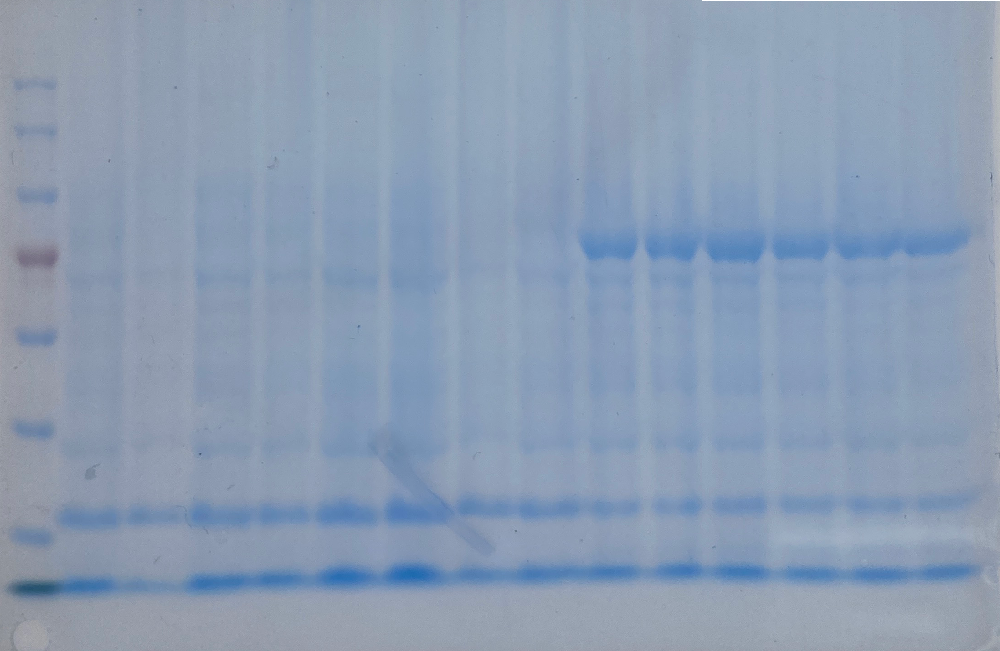

Supplement: Supplementary file 1 [file plants-15-01057-s001.zip › sig2a protein.tif]

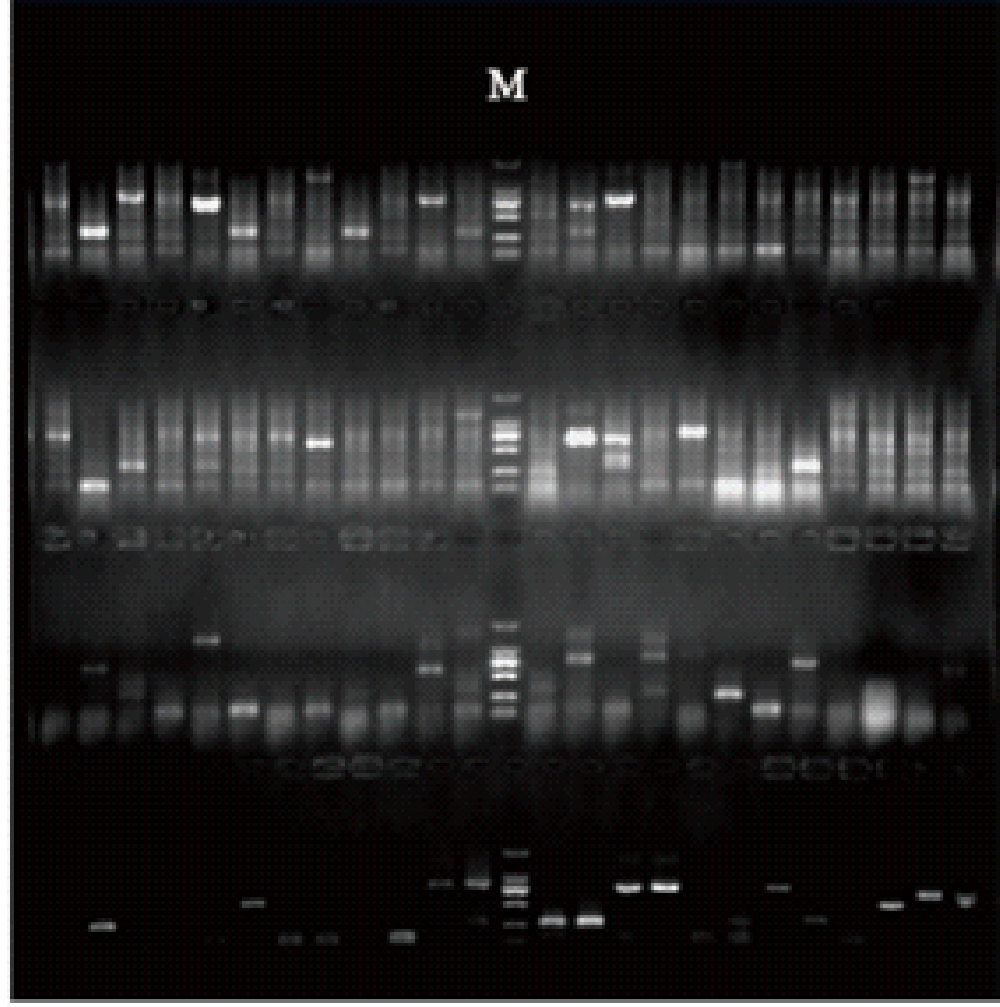

Supplement: Supplementary file 1 [file plants-15-01057-s001.zip › Y1H gels .tif]
